# Nerve ECM and PLA-PCL based electrospun bilayer nerve conduit for nerve regeneration

**DOI:** 10.3389/fbioe.2023.1103435

**Published:** 2023-03-02

**Authors:** Xiaoyan Mao, Ting Li, Junqiu Cheng, Meihan Tao, Zhiyuan Li, Yizhan Ma, Rabia Javed, Jie Bao, Fang Liang, Weihong Guo, Xiaohong Tian, Jun Fan, Tianhao Yu, Qiang Ao

**Affiliations:** ^1^ Department of Tissue Engineering, China Medical University, Shenyang, China; ^2^ Department of Laboratory Medicine, Shengjing Hospital of China Medical University, Shenyang, China; ^3^ National Engineering Research Center for Biomaterials, Sichuan University, Chengdu, China; ^4^ Fuwai Hospital, Chinese Academy of Medical Sciences and Peking Union Medical College, Beijing, China; ^5^ Liaoning Provincial Key Laboratory of Oral Diseases, The VIP Department, School and Hospital of Stomatology, China Medical University, Shenyang, China; ^6^ Institute of Regulatory Science for Medical Device, Sichuan University, Chengdu, China

**Keywords:** peripheral nerve regeneration, electrospun, ECM, bilayer structure, nerve conduit

## Abstract

**Introduction:** The porcine nerve-derived extracellular matrix (ECM) fabricated as films has good performance in peripheral nerve regeneration. However, when constructed as conduits to bridge nerve defects, ECM lacks sufficient mechanical strength.

**Methods:** In this study, a novel electrospun bilayer-structured nerve conduit (BNC) with outer poly (L-lactic acid-co-ε-caprolactone) (PLA-PCL) and inner ECM was fabricated for nerve regeneration. The composition, structure, and mechanical strength of BNC were characterized. Then BNC biosafety was evaluated by cytotoxicity, subcutaneous implantation, and cell affinity tests. Furthermore, BNC was used to bridge 10-mm rat sciatic nerve defect, and nerve functional recovery was assessed by walking track, electrophysiology, and histomorphology analyses.

**Results:** Our results demonstrate that BNC has a network of nanofibers and retains some bioactive molecules, including collagen I, collagen IV, laminin, fibronectin, glycosaminoglycans, nerve growth factor, and brain-derived neurotrophic factor. Biomechanical analysis proves that PLA-PCL improves the BNC mechanical properties, compared with single ECM conduit (ENC). The functional evaluation of *in vivo* results indicated that BNC is more effective in nerve regeneration than PLA-PCL conduit or ENC.

**Discussion:** In conclusion, BNC not only retains the good biocompatibility and bioactivity of ECM, but also obtains the appropriate mechanical strength from PLA-PCL, which has great potential for clinical repair of nerve defects.

## 1 Introduction

Severe peripheral nerve injuries often lead to partial or total loss of motor and/or sensory functions associated with neuropathic pain. It affects the life quality of millions of people each year worldwide and results in an enormous medical and economic burden for society. Despite the rapid advancements in microsurgical techniques, complete functional recovery following peripheral nerve repair has not been well achieved (Sachanandani et al., 2014; Navarro, 2016).

Autologous nerve grafting (ANG) is considered the gold standard for bridging peripheral nerve defects (Carvalho et al., 2017; Wieringa et al., 2018; Zhang et al., 2018; Javed and Ao, 2022). The success of the treatment is largely attributed to the presence of Schwann cells (SCs) which provide neurotrophic factors, the basal lamina endoneurial tubes, and the endoneurial tube surface adhesion molecules which are capable of enhancing axon regeneration (Choi et al., 2018). However, its disadvantages include secondary surgery, limited donor nerve source, functional loss of the donor sites, painful neuroma formation, and neurological dysfunction (Hoben et al., 2018; Han et al., 2019). Moreover, there is a frequent size mismatch of donor nerves, which greatly limits their clinical application (Lee and Wolfe, 2000). To address these obstacles, various clinically available commercial nerve conduits have been developed, such as biodegradable nerve conduits derived from synthetic polymers and natural biopolymers (Sanchez Rezza et al., 2022). Synthetic polymers include polyurethane, polyglycolic acid, polycaprolactone, and poly (lactic acid-co-glycolic acid) (Wilson et al., 1983; Liu et al., 2008; Panahi-Joo et al., 2016; Kusuhara et al., 2019). Natural biopolymers are mainly collagen, chitosan, alginate, gelatin, or silk fibroin (Büyüköz et al., 2018; Gnavi et al., 2018; Nune et al., 2019; Zhang et al., 2021). The above-mentioned nerve conduits only provide a relatively isolated environment for nerve regeneration, but lack either biocompatibility or sufficient mechanical strength, which results in slow nerve regeneration (Wang et al., 2012a; Büyüköz et al., 2018; Han et al., 2019). Therefore, a novel nerve repair conduit is urgently needed.

Recently, researchers found that nerve-derived extracellular matrix (ECM) had good performance in nerve repair (Ren et al., 2018; Schaeffer et al., 2018; Chen et al., 2019; Han et al., 2019; Li et al., 2021). The most commonly used ECM is derived from decellularized porcine nerves (DPNs) due to its similarity to human nerve in the anatomical, biochemical, and molecule components. ECM elicits low stimulation to the host immune system and contains different kinds of functional proteins, including collagens, proteoglycans, glycosaminoglycans, and growth factors, which could promote SCs proliferation, migration, or differentiation (Li et al., 2020). Zilic et al. (2016) have demonstrated that SCs cultured in contact with DPN could not only survive but also proliferate and retain their phenotype. In addition, studies have found that the conduits consisting of a collagen wall and nerve ECM inside are more effective for peripheral nerve regeneration than the silicone conduit (Choi et al., 2018). Various studies have proved nerve ECM an ideal nerve repair material. However, our previous study showed that the DPNs-derived conduits lack sufficient mechanical support and thus are susceptible to compression from surrounding tissues (Sun et al., 2018).

Poly (L-lactic acid-co-ε-caprolactone) (PLA-PCL) is a synthetic co-polymer of poly (L-lactic acid) (PLLA) and poly (ε-caprolactone) (PCL) (Song et al., 2016). As a linear aliphatic polyester with the ability to be resorbed and eliminated through the citric acid cycle, PLA-PCL is biodegradable and bioabsorbable (Jeong et al., 2005; Wang et al., 2013). Its tailorable properties through composition and molecular weight offer their mechanical properties and degradation profile suitable for biomedical applications. Generally speaking, PLA-PCL is highly elastic and non-toxic (Song et al., 2016). Compared with single PCL or PLLA, PLA-PCL has superior mechanical properties and biodegradability for the applications in bridging damaged nerve gaps (Jeong et al., 2005; Wang et al., 2013; Zhang et al., 2020). While PLA-PCL are biocompatible, they do not contain the ingredients capable of promoting nerve growth, some improvement should be made to favor a functional and effective nervous regeneration. Incorporation of nerve ECM seems to be a possible way to afford biological cues, which, however, has not been reported too much.

Solution electrospun is a simple and inexpensive technique extensively used in producing polymeric nanofibrous meshes. Especially, electrospun technique can both introduce mechanical anisotropy and provide topographic features to the fabricated structures, such like the controlled surface morphology, the diameters ranging from micro-to nano-meter, high surface area to volume ratio, low density, and high porosity. The electrospun polymeric nanofibers thus mimic a native-like microenvironment of nerve to some extent that would favor SCs proliferation, adhesion, and axon growth (Wang et al., 2013; Singh et al., 2020), which converts electrospun as an ideal technology for nerve regeneration.

A successful nerve conduit will not only provide contact guidance for axonal extension but also afford a wall robust and tear-resistant enough to withstand the surgical operation. It is optimal to combine the advantages of both electrospun PLA-PCL and nerve ECM so as to improve the mechanical properties and bioactivities of DPNs conduits. To this end, a novel bilayer-structured nerve conduit (BNC) would be developed by electrospun the PLA-PCL as the outer layer and ECM as the inner layer, with the outer PLA-PCL layer offering enough mechanical strength and the inner ECM layer promoting nerve regeneration.

In this study, the novel BNC electrospun from PLA-PCL and ECM was fabricated for nerve regeneration. The BNC had a suitable size, sufficient mechanical support, good biocompatibility, and a network of nanofibers. The components of BNC were tested by immunohistochemistry, enzyme-linked immunosorbent assay (ELISA), and western blot. The mechanical properties of BNC were performed on a tensile-testing machine, and its degradability was tested by a degradation rate test *in vitro*. The cytotoxicity assay, subcutaneous implantation test, and cell affinity assay were performed to evaluate the biocompatibility of BNC. Furthermore, BNC was implanted in a 10-mm defect in a rat sciatic nerve model for up to 12 weeks in comparison with the PLA-PCL conduit (PPC), the ECM conduit (ENC), and ANG. The treatment efficacy of nerve regeneration was evaluated by walking track analysis, electrophysiological assay, and histological analysis.

## 2 Methods

### 2.1 Materials

PLA-PCL (50:50) was purchased from Jinan Dai Gang Biological Co., Ltd. (China, Mw: 1.10 × 10^5^). Glacial acetic acid (> 99.70% pure) was purchased from Sigma-Aldrich, United States. Dulbecco’s modified Eagle’s medium (DMEM), fetal bovine serum (FBS), penicillin/streptomycin, and 0.25% of trypsin-EDTA phenol red were purchased from Gibco, United States. Triton X-100 and sodium deoxycholate were purchased from Sigma Chemical Company (United States).

### 2.2 Preparation of decellularized porcine sciatic nerve

FPNs were harvested from crossbred pigs (12 months old) at the slaughterhouse according to ISO 22442-2. Excess fat and connective tissue were dissected from the nerve samples. The FPNs were thoroughly washed with deionized water and cut into 2 cm pieces before decellularization. Then, the nerve samples were physically decellularized, frozen at −80°C for no less than 6 h and thawed in a 37°C water bath for about 30 min. The freeze-thaw cycle was repeated 3 times. Next, the nerve samples were rinsed with deionized water under agitation at room temperature for 6 h. Finally, the samples were treated with chemical decellularized reagents, 3% (v/v) Triton X-100 (Sigma, United States) for 12 h and 4% (w/v) sodium deoxycholate (Sigma, United States) for 24 h, and after each of these steps, washing with deionized water thoroughly under constant vibration (100 rpm) in a plate shaker, 3 times for 15 min each. The resulted DPNs were frozen and stored at −80°C in phosphate buffered saline (PBS) until use. Hematoxylin and eosin (HE) staining and Genomic DNA Extraction Kit (Tiangen, China) were performed to analyze the decellularization efficiency of the porcine sciatic nerves.

### 2.3 Preparation of DPN derived ECM solution and PLA-PCL solution for electrospun

Briefly, the DPN was cut into slices with 1 μm–2 μm thickness using a freezing microtome (Thermo, United States). After lyophilization, 0.50 g of DPN slices were completely dissolved in 5 mL of acetic acid under constant stirring at 300 rpm for 48 h until the formation of an ivory creamy homogeneous system. The concentration of the ECM was 10% (W/V). Similarly, 0.50 g of the copolymer PLA-PCL powders were dissolved in 5 mL of acetic acid at room temperature for 8 h. The concentration of PLA-PCL was 10% (W/V). Both the ECM and PLA-PCL solutions were sonicated to remove the air bubbles.

### 2.4 Construction of three kinds of nerve conduits

#### 2.4.1 Construction of PPCs

10 wt% solution of PLA-PCL was loaded into a 5 mL plastic needleless injector. The polymer solution was sprayed with a metal spinneret (G20) to a metal collecting iron bar. The parameters of the electrospun condition were as follows: the positive voltage of 8 kV, the negative voltage of −3 kV, the flow rate of 0.20 mL/h, the syringe distance of 18 cm, the height of 34 cm, the collector rotating rate of 500 r/min, the electrospun temperature of 20°C–25°C, and the electrospun humidity of 50%–60%. A metallic collector iron bar covered with aluminum foil was used to avoid any fiber damage while removing the conduit. The electrospun time was 4 h in order to prepare the final PPCs with suitable hardness.

#### 2.4.2 Construction of ENCs

10 wt% solution of ECM was loaded into a 5 mL plastic needleless injector with a metal spinneret (G20). The parameters of the height, the collector rotating rate, the electrospun temperature, and the electrospun humidity were the same as those for the construction of PPCs. The difference was that the positive voltage was 12 kV and the negative voltage was −2.50 kV. The ENCs were removed from the metal collecting iron bar and cut to a length of 10 mm. The electrospun time was 8 h.

#### 2.4.3 Construction of BNCs

Briefly, a 10 wt% solution of ECM was first electrospun and collected on a metal collecting iron bar as the inner layer of the conduit. The electrospun time was 6 h. Then a 10 wt% solution of PLA-PCL was electrospun as an outer layer of the conduit for 2 h. The parameters were the same as those for the construction of PPCs and ENCs.

#### 2.4.4 Pretreatment before implantation

The inner diameter of the nerve conduits is 1.60 mm, which matches the diameter of the rat sciatic nerve. Before implantation, 3 group nerve conduits were subjected to 0.1 N NaOH to adjust the pH to 7.4 and then thoroughly rinsed with deionized water for 30 min to remove any residual solvents. After drying in a vacuum oven at room temperature for 24 h, the prepared conduits were sterilized with cobalt-60.

### 2.5 Scanning electron microscopy (SEM) assessment

The microstructure morphologies of the BNC and ENC were observed by SEM. First, the samples were fixed with 2.50% glutaraldehyde (Sigma) for 6 h at 4°C, followed by washing in PBS 3 times (5 min each) and dehydration in a graded series of ethanol (30%, 50%, 70%, 80%, 90%, and 100% ethanol for 30 min each). Later on, the samples were rinsed with deionized water 3 times (1 h each) and then lyophilized for 12 h. A thin layer of platinum alloy film was coated onto the surface of the conduits before observation. Finally, the samples were observed with a VEGA3 tungsten scanning electron microscope (TESCAN, Czech) at an accelerating voltage of 10 kV.

### 2.6 Composition analysis

#### 2.6.1 Histological analysis

FPN, DPN, and ENC samples were fixed in 4% paraformaldehyde solution for 24 h. After dehydration with graded ethanol and hyalinization with xylene, the samples were embedded in paraffin and sectioned into 8 μm slices. The slices were dried, deparaffinized, rehydrated, and washed in distilled water. Then, the sections were stained and observed under a microscope. HE staining was used to observe the structure of FPN, DPN, and ENC, and to identify the presence of residual cell components. 4′,6-diamidino-2-phenylindole (DAPI, Sigma-Aldrich, United States) staining was performed to confirm the absence of the residual nucleus. In addition, Masson trichrome staining was used for the morphological characterization of collagen fibers.

#### 2.6.2 DNA quantification

DNA quantification was used to detect the total DNA contents of FPN, DPN, and ENC. DNA extraction was carried out using a genomic DNA extraction kit (Tiangen, China) following the manufacturer’s instructions. Firstly, the 3 group samples were collected and cut into slices with a freezing microtome. Then, the 3 group samples were lyophilized to a constant weight with a vacuum freeze drier. Next, 3 group samples weighing approximately 25 mg were digested with Lysis Buffer, Proteinase k at 56°C till complete digestion, and then transferred to the centrifugal columns. The DNA components absorbed on the silicon substrate membranes were finally dissolved in the TE buffer. Lastly, the concentration of the extracted DNA was measured using a microplate spectrophotometer, and the size of the extracted DNA fragments was detected by 1% agarose gel electrophoresis. Five samples were tested for each group.

#### 2.6.3 Total collagen quantitative detection

The collagen contents in FPN, DPN, and ENC were quantified using a hydroxyproline assay kit (Nanjing Jiancheng Bioengineering Institute, China). Following the manufacturer’s instructions, 3 group samples were cut into slices and lyophilized to a constant weight. Then, 3 group samples weighing approximately 25 mg each by dry weight were mixed with 6 M hydrolysates (HCL) at 95°C for over 5 h. Next, the pH value of 3 samples was adjusted to 6.0–6.8 using 6 M HCL. The supernatant was collected and mixed with the reagents of the assay. Finally, the absorbance was read at 570 nm using a microplate spectrophotometer. The total collagen content was calculated according to the hydroxyproline-to-collagen ratio of 1:7.69 (Zilic et al., 2016). Five samples were detected for each group.

#### 2.6.4 Glycosaminoglycans (GAGs) quantitative detection

The contents of GAGs in FPN, DPN, and ENC were detected and quantified by a dimethylmethylene blue assay (GenMed Scientific INC, United States). Briefly, following the manufacturer’s instructions, 3 group samples were collected and cut into slices, incubated in water at 56°C for 16 h, and then at 90°C for 10 min. The supernatant was collected and added to a clear flat-bottomed 96-well plate. Finally, the absorbance values were measured at 570 nm by using a microplate spectrophotometer. Five samples were tested for each group.

#### 2.6.5 Immunohistochemistry

To compare the effect of decellularization process on the preservation of main bioactive molecules of ECM, collagen I, collagen IV, laminin (LN), and fibronectin (FN) in FPN and DPN were detected by immunohistochemistry. Briefly, the samples were fixed in 4% paraformaldehyde solution for 24 h. After dehydration with graded ethanol and hyalinization with xylene, the samples were embedded in paraffin and sectioned into 8 μm slices. Next, the IHC Kit (Mouse anti-Rabbit, MXB^®^ Biotechnologies, China) was used according to the manufacturer’s instructions. The slices were incubated in 3% (v/v) H_2_O_2_ for 10 min to block endogenous peroxidase activity. Non-specific staining was blocked with non-immune serum for 30 min, followed by overnight incubation at 4°C with anti-collagen I, anti-collagen IV antibodies, anti-laminin, and anti-fibronectin (1:200, Abcam, United States). The secondary antibody (Mouse anti-Rabbit, MXB^®^ Biotechnologies, China) was applied for 30 min after washing in PBS 3 times. Subsequently, the slices were stained with 0.02% diaminobenzidine for 5 min (MXB^®^ Biotechnologies, China) and counterstained with hematoxylin. Finally, the slides were dehydrated, cleared, mounted, and visualized with an Olympus BX53 (Japan) light microscope.

#### 2.6.6 Western blot analysis for main bioactive molecules of ECM

To determine the impact of the electrospun process on the preservation of the main bioactive molecules of ECM, and whether ENC retained ECM-related components, collagen I, collagen IV, LN, and FN were detected in DPN and ENC by immunoblotting. Briefly, the total proteins of the DPN and ENC groups were extracted using the protein extracted kit (Keygen, China). After centrifuging at 12,000 g and 4°C for 30 min, the supernatant was collected. The concentration was measured using a BCA protein assay (Beyotime Biotechnology, China). All the samples were mixed with sodium dodecyl sulfate-polyacrylamide gel electrophoresis (SDS-PAGE) loading buffer and boiled for 5 min. Equal amounts of protein (20 µg) were electrophoretically separated on two 8% SDS-PAGE gels. The one used as a control for loading was stained with Coomassie Brilliant Blue R-250 for 2 h, rinsed overnight, and imaged. The total protein was quantified using ImageJ software (National Institutes of Health, United States). The other gel was transferred to a polyvinylidene difluoride membrane and blocked with 5% non-fat milk for 1 h. Subsequently, the membranes were incubated overnight at 4°C with anti-laminin, anti-fibronectin, anti-collagen I, or anti-collagen IV antibodies (all at 1:1,000 dilution and all from Abcam, United States), and then incubated with horseradish peroxidase-conjugated anti-rabbit secondary antibody (1:5,000, Abcam, United States) for 2 h at room temperature. The protein bands were visualized with the enhanced chemiluminescence kit (Tiangen Biotech, China) and quantified by grayscale analysis using ImageJ software.

#### 2.6.7 ELISA for main bioactive molecules of ECM

To evaluate the impact of the decellularization process and electrospun process on the preservation of the main bioactive molecules of ECM, an ELISA was carried out to quantify the contents of collagen I, collagen IV, LN, and FN in FPN, DPN, and ENC. Total proteins of the FPN, DPN, and ENC groups were extracted using the protein extraction kit (Keygen, China). After centrifuging at 12,000 g and 4°C for 30 min, the supernatant was collected. The concentration was measured using a BCA protein assay (Beyotime Biotechnology, China). The ELISA kit (USCN Life ScienceTechnology, China) was used to quantify the protein contents of 3 group samples following the manufacturer’s instructions. First, the supernatant of the 3 groups was sonicated with an ultrasonic cell disrupter till the solution was clarified. Then, 100 μL of samples were added to the 96-well plates in triplicate and mixed gently to avoid foaming. Next, the samples were incubated for 2 h at 37°C. Finally, a microplate spectrophotometer was used to analyze the absorbance values of each well at 450 nm. The standard curve was plotted with the data produced from the diluted standard solutions. The concentrations of collagen I, collagen IV, LN, and FN in the 3 groups were calculated according to the standard curve. The final concentration was expressed as the proportion of collagen I, collagen IV, LN, and FN in the total protein, respectively. All detections were independently repeated five times.

#### 2.6.8 ELISA for critical regenerative factors

To investigate the impact of the decellularization and electrospun on the preservation of the critical regenerative factors, an ELISA was carried out to detect the nerve growth factor (NGF) and brain-derived neurotrophic factor (BDNF) in FPN, DPN, and ENC. The ELISA kit (Nanjing Jiancheng Bioengineering Institute, China) was used to analyze the contents of these factors in the extracted supernatant according to the manufacturer’s instructions. The standard samples and protein extracts of FPN, DPN, and ENC (100 μL) were added to the wells in triplicate and incubated at 37°C. The absorbance of each well was examined at 450 nm. The standard curve was plotted with the data produced from the diluted standard solutions. The concentrations of NGF and BDNF were calculated based on the appropriate standard curve, and the final concentration was expressed as the proportion of the NGF and BDNF in the total protein, respectively.

### 2.7 Mechanical property

The values of the max suture retention strength of the conduits in ENC (*n* = 5), BNC (*n* = 5), and PPC (*n* = 5) were tested with an electric dynamic test system (Shanghai, China). The samples (10 mm in length) were soaked in 0.1 mol/L PBS before testing.

To determine the max suture retention strength of the conduits-epineuria, every conduit (10 mm length) was sutured between the 2 stumps of the porcine sciatic nerves with 8-0 nylon, the suture was pierced through epineurium or conduits at 1 mm from the edge, with 4 stitches evenly distributed on the edge of the nerve or conduit ends. At least 4 knots were tied at the end of each suture to prevent slippage. The 2 nerve stumps were clamped by the fixtures of the testing device and stretched at a rate of 10 mm/min until the suture was pulled out of the conduits or epineuria. The maximum load at the breaking point was recorded as the max suture retention strength of the conduits-epineuria.

After the suture was pulled out of the epineuria, the max suture retention strength of the conduits was further measured. The conduit was pierced through with 8-0 nylon with the other side of the suture clamped by the testing device. The force was applied parallel to the axis of the conduits at an extension speed of 10 mm/min until the suture was pulled out of the conduits. The maximum load at the breaking point was recorded as the max suture retention strength of the conduits.

To determine the max suture retention strength of the natural epineuria, the 2 stumps of the porcine sciatic nerves were sutured with 8-0 nylon. The suture was pierced through the epineurium at 1 mm from the edge, with 4 stitches evenly distributed on the edge of the nerve ends. At least 4 knots were tied at the end of each suture to prevent slippage. The 2 nerve stumps were clamped with the fixtures of the testing device and stretched at a rate of 10 mm/min until the suture was pulled out of the epineurium. The maximum load at the breaking point was recorded as the max suture retention strength of the natural epineurium.

### 2.8 Water absorption analysis

The swelling behavior of the conduits was tested to assess the water absorption of each group (*n* = 6), which also reflected the porosity and hydrophilicity. The samples were immersed in PBS at 4°C for 24 h to become fully swollen, and then lyophilized for about 12 h to obtain a constant dry weight (Wd). Subsequently, the lyophilized samples were immersed in PBS at 4°C for 24 h to achieve a constant swelling weight (Ws). The water weight was calculated by subtracting the dry weight from the swelling weight. The swelling ratio was calculated with the formula $\left(Ws-Wd\right)/Wd$ and expressed as %.

### 2.9 *In vitro* degradation

To determine the degradation of the 3 conduits, the samples (*n* = 6 for each group) were lyophilized for about 12 h, then pre-weighed (W1) and soaked in 0.1 mol/L PBS (pH =7.20-7.40) at 37°C. The degradation solution was replaced with fresh PBS solution every week. At each week, the nerve conduits were taken out from PBS and weighed (W2) after lyophilization. This process was repeated four times. The degradation (%) was calculated using the following equation (Askarzadeh et al., 2020):

$$\text{Degradation}\left(\%\right)=\frac{\left(W1-W2\right)}{W1}$$ (1)

### 2.10 Biocompatibility of three kinds of conduits

#### 2.10.1 Cytotoxicity assay

The cytotoxicity of 3 group conduits to SCs was evaluated using the Cell Counting Kit-8 (CCK-8). The samples of PPCs, BNCs, and ENCs were placed into 48-well tissue culture plates respectively and sterilized by 75% ethanol and exposure to UV irradiation overnight. Then the ethanol was removed by rinsing with deionized water thoroughly (3 times for 30 min each). The extract mediums of the 3 group samples were prepared according to the International Standard ISO 10993-12. Briefly, the conduits were incubated in 1 mL DMEM at 37°C for 24 h, and then the degraded debris was filtered and the extract mediums were collected. In addition, a normal medium control (DMEM), a negative control (polyethylene), and a 10% (v/v) solution of dimethyl sulfoxide were prepared as toxicity positive control. SCs were cultured in complete DMEM with 10% FBS and 1% penicillin/streptomycin, as described previously (Hou et al., 2018). And then the SCs pellets were seeded at a density of 2 × 10^4^ cells/well on 96-well tissue culture polystyrene plates. After the cell adherence, the medium was replaced by the sample extracts of different concentrations (25%, 50%, 75%, and 100%). The 96-well plates were transferred to incubator at 37°C, 5% CO_2_, and 95% humidity. The cell proliferation was evaluated on the 3rd day. After refreshing the medium, 10 µL of CCK-8 solution was added to each well followed by incubation for 2 h at 37°C. And finally, the absorbance values were measured at 450 nm using a microplate spectrophotometer. 6 parallel experimental groups for each sample were set. The cell proliferation (%) was calculated according to the formula:

$$\text{Cell}\;\text{proliferation}\left(\%\right)=\frac{OD\;test}{\text{OD}\;\text{medium}}$$ (2)

where OD test is the absorbance of each group and OD medium is the absorbance of DMEM group.

#### 2.10.2 Cell affinity evaluation

SEM was used to observe the SCs cultured on the surface of 3 group nerve conduits. Briefly, PPCs, ENCs, and BNCs were cut into square shapes (0.50 cm × 0.50 cm) to fit the bottom area of the well in the 24-well plate. Three group conduits were placed into a 24-well culture plate and incubated in 1 mL complete DMEM medium with 10% FBS and 1% penicillin/streptomycin. After 24 h, the SCs at a density of 2 × 10^4^ cells/cm^2^ were seeded on the surface of 3 group nerve conduits and then incubated in 1 mL media. After 3 days, 3 group conduits were taken out and fixed with 2.50% glutaraldehyde (Sigma, United States) solution for 24 h. Next, after the three group conduits were washed with PBS (3 times for 15 min each), the conduits were dehydrated with graded ethanol (30%, 50%, 60%,70%, 80%, 90%, and 100% ethanol for 15 min each), and lyophilized by vacuum freeze drier. Finally, the conduits were coated with gold and a JSM 7001F tungsten scanning electron microscope (JEOL, Japan) was used to observe the cells on the sample surface.

#### 2.10.3 Subcutaneous implantation tests

The animal experiment was performed according to the protocols approved by the Institutional Animal Care and Use Committee at China Medical University and the Local Ethical Committee for Laboratory Animals. Different samples, including BNCs, ENCs, and PPCs, were cut into shapes (1 cm × 1 cm). Before implantation, 3 group conduits were immersed in 75% of medical alcohol for 30 min for sterilization and then randomly used to implant subcutaneously in a total of 27 healthy male Wistar rats (180 g–200 g). The rats were anesthetized by intraperitoneal injection of 1% sodium pentobarbital solution (40 mg/kg body weight) and shaved back hair and sterilized. The back incision was located at the right side about 1.50 cm–2 cm away from the central line, then the submucosa was separated from the right to the left, and the materials were implanted into the back. The 5-0 nylon sutures were used to close muscle and skin. After operation, the rats were housed under standard conditions. And then the rats were euthanized using pentobarbital sodium at 1, 4, 12 weeks after surgery and the 3 group conduits with adjacent tissues were cut off and fixed in 4% paraformaldehyde. Finally, HE staining was conducted to demonstrate the changes in inflammatory cell numbers and distribution after implantation. Immunohistochemical staining was used to observe the changes in the density and distribution of CD68-positive cells around and within the implanted conduits. The numbers of the nuclei and stained cells were counted using ImageJ software. The number of CD68-positive cells was counted at 400 × and averaged over 5 areas per sample.

### 2.11 Repair of sciatic nerve defects with the 3 kinds of conduits

#### 2.11.1 Surgical procedures

24 healthy male Wistar rats weighing 180 g–200 g were randomly divided into 4 experimental groups, including the BNCs group (*n* = 6), ENCs group (*n* = 6), PPCs group (*n* = 6), and ANGs group (*n* = 6) for *in vivo* nerve repair studies. All protocols were approved by the Institutional Animal Care and Use Committee at China Medical University. The rats were anesthetized by intraperitoneal injection of 1% sodium pentobarbital solution (40 mg/kg body weight). Under the posterior operation, the left sciatic nerve was exposed. For the conduit groups, a 10 mm nerve conduit was used to link the two severed nerve ends. 1 mm of nerve stumps were inserted into the conduit and fixed with 8-0 nylon sutures, maintaining an 8 mm nerve gap. For the ANG group, a 10 mm segment of the sciatic nerve was transected, and the removed nerve segment was reversed and sutured to the proximal and distal nerve ends. The epineuria of the proximal and distal nerve ends were sutured by 8-0 nylon sutures, and the muscle and skin were closed with 5-0 nylon sutures. After surgery, the rats were fed and housed separately for 12 weeks.

#### 2.11.2 Sciatic function index (SFI)

12 weeks after the surgery, a walking track analysis was performed to evaluate the functional muscle reinnervation as described previously (Navarro, 2016). Preoperatively, the rats were painted with black ink on the plantar surfaces of both hind paws and allowed to walk along a narrow corridor with paper towards a dark compartment at the end. Footprints obtained without surgery were considered normal feet (N), and those obtained with surgery were experimental feet (E). PL (the length of the footprint from the third toe to the heel) reflected gastrocnemius activation, and TS (the toe spread distance between the first and the fifth toe) and IT (the toe spread distance between the second and the fourth toe) were mainly influenced by paw extensor and paw intrinsic muscle contraction during the stance phase of walking. The SFI was calculated according to the following formula described by Bain et al. (Wang et al., 2012b):

$$SFI=38.3[\left(EPL-NPL\right)÷NPL]+109.5\left[\left(ETS-NTS\right)÷NTS\right]+13.3[\left(EIT-NIT\right)÷NIT-8.8.$$ (3)

SFI oscillates around 0 or around −100, which represents normal nerve function or complete loss of function.

#### 2.11.3 Muscle recovery evaluation

After nerve reinnervation was established, the atrophied muscle would gradually recover. The circumferences of the mid-shank of hind limbs on the injured side and normal side were measured to assess the effects of the 3 conduits on muscle recovery post-operation. The acquired data were expressed as the circumference ratio between the injured side and the normal side.

#### 2.11.4 Electrophysiological testing

Electrophysiological analyses were performed to assess motor axon regeneration and muscle reinnervation at 12 weeks postoperatively. Briefly, 6 rats from each group were re-anesthetized with 1% sodium pentobarbital solution (40 mg/kg body weight), and the nerve-repaired site was re-exposed. A multichannel electrophysiological system (RM6240, China) was used with the bipolar stimulating electrodes attached to the proximal and distal ends of the nerve repair site respectively, and a grounding electrode inserted into the skin in the femoral region. The nerve was stimulated by increasing the intensity of the electrical pulses till the reaction occurred. Then the compound muscle action potential (CMAP) was recorded by the recording electrode inserted in the triceps in crus. The motor nerve conduction velocity (MCV) was determined according to the conduction time of the impulse along the nerve and the nerve distance between the proximal and distal stimulation sites. The CMAP and MCV were examined at the stimulation mode (stimulus intensity = 6 mA; frequency = 1 Hz; duration = 1 ms) and measured by the PowerLab device and software. The acquired data of CMAP amplitude were expressed as the ratio between the injured side and the normal side to eliminate variations between rats.

#### 2.11.5 Toluidine blue staining of the myelin sheath

Following the electrophysiological analyses, the distal segments near the ends of the nerve conduits from each group were prefixed in 4% paraformaldehyde solution for 48 h and postfixed in 1% osmium tetroxide for 1.50 h. After a series of dehydration, the tissue samples were embedded in Epon 812 epoxy resin and cut into 0.50 μm thick sections using glass knives. Then the obtained sections were stained with 1% toluidine blue solution (Solarbio, China) and observed under a light microscope (BX53; Olympus, Japan). To count the number of myelinated axons, 10 high-power fields (HPF, × 400) from each sample were randomly selected. The mean density of myelinated axons (number of axons/mm^2^) of each sample was quantified using ImageJ software. Both fiber and axon area were measured and the diameter of fiber (D) and axon (d) were calculated. These data were used to calculate myelin thickness [(D-d)/2], myelin thickness/axon diameter ratio [(D-d)/2d], and axon/fiber diameter ratio, the g-ratio (D/d) (Ronchi et al., 2014).

#### 2.11.6 Immunofluorescence histopathology

After the electrophysiological analysis, the distal segments near the ends of the nerve conduits from each group were fixed in 4% paraformaldehyde solution for 12 h, soaked in 30% sucrose solution at 4°C for 24 h until the samples sank to the bottom of the container. Then the tissue samples were frozen and cut into 8 μm–10 μm sections with a freezing microtome. The obtained sections were transferred onto glass slides and incubated in the PBS containing 1% normal goat serum for 1 h at 24°C. After that, the sections were incubated overnight at 4°C with anti-neurofilament antibody (anti-NF200, 1:200 dilution; Abcam, United States) and anti-S100 antibody (1:200 dilution; Abcam, United States), followed by the incubation for 1 h at room temperature with secondary antibodies (goat anti-rabbit IgG antibody and goat anti-mouse IgG antibody at 1:1,000 dilution; Abcam, United States). Images were acquired by a laser confocal microscope (FV1200; Olympus, Japan).

### 2.12 Statistical analysis

All quantitative data were expressed as means ± standard errors of the mean (SD). All collected data were analyzed using GraphPad Prism 5.0 software (United States). All differences were calculated using one-way analysis of variance (ANOVA) or unpaired *t-*tests. In all cases, the level of *p* < 0.05 was recognized as significantly different.

## 3 Results

### 3.1 BNC conduit fabrication and surface characterization

**FIGURE 1:**
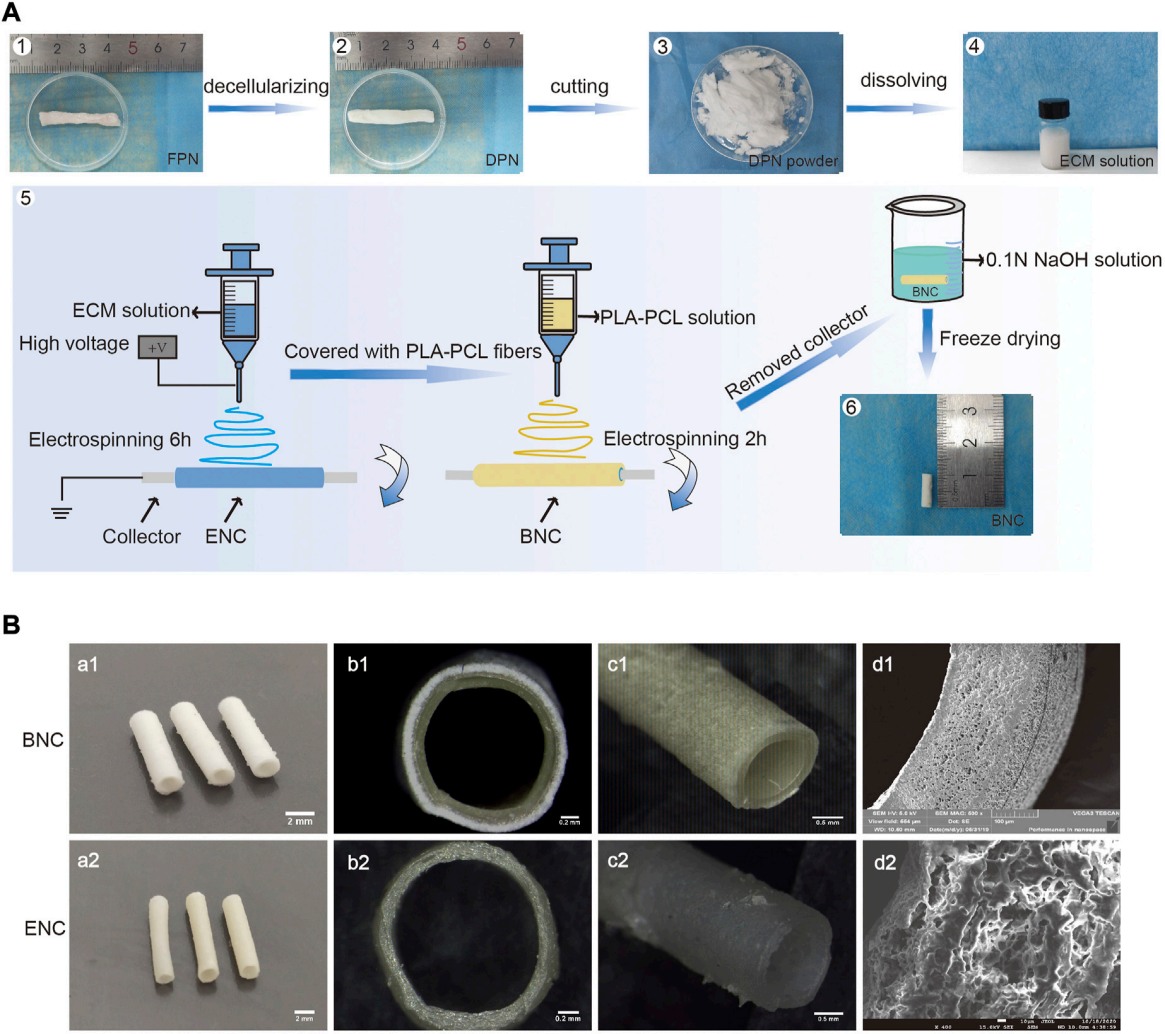
The fabrication of BNC. **(A)** The procedure of manufacturing BNC. i.e., briefly, the fresh porcine sciatic nerves (FPNs) were thoroughly decellularized to DPNs, cut into slices, lyophilized to powders, completely dissolved into the ECM solution, and lastly processed to BNC by electrospun. **(B)** The SEM and the stereoscope images of BNC and ENC.

The procedure for manufacturing BNC is shown in Figure 1A. The BNC has a wall thickness of approximately 0.222 mm ± 0.018 mm including 0.121 mm ± 0.010 mm thick PLA-PCL and 0.104 mm ± 0.008 mm thick ECM, and an inner diameter of BNC about 1.342 mm ± 0.025 mm (Figure 1B). The SEM images and the stereoscope images show that the BNC is a double-layer structure, and there is no significant separation between the 2 layers (Figures 1B–b1, d1). SEM observation reveals that the BNC has a fibrous inner layer and outer layer with a nanofibrous and porous structure, which facilitates cell infiltration and migration (Jin et al., 2019).

### 3.2 Confirmation of decellularization effectiveness

**FIGURE 2:**
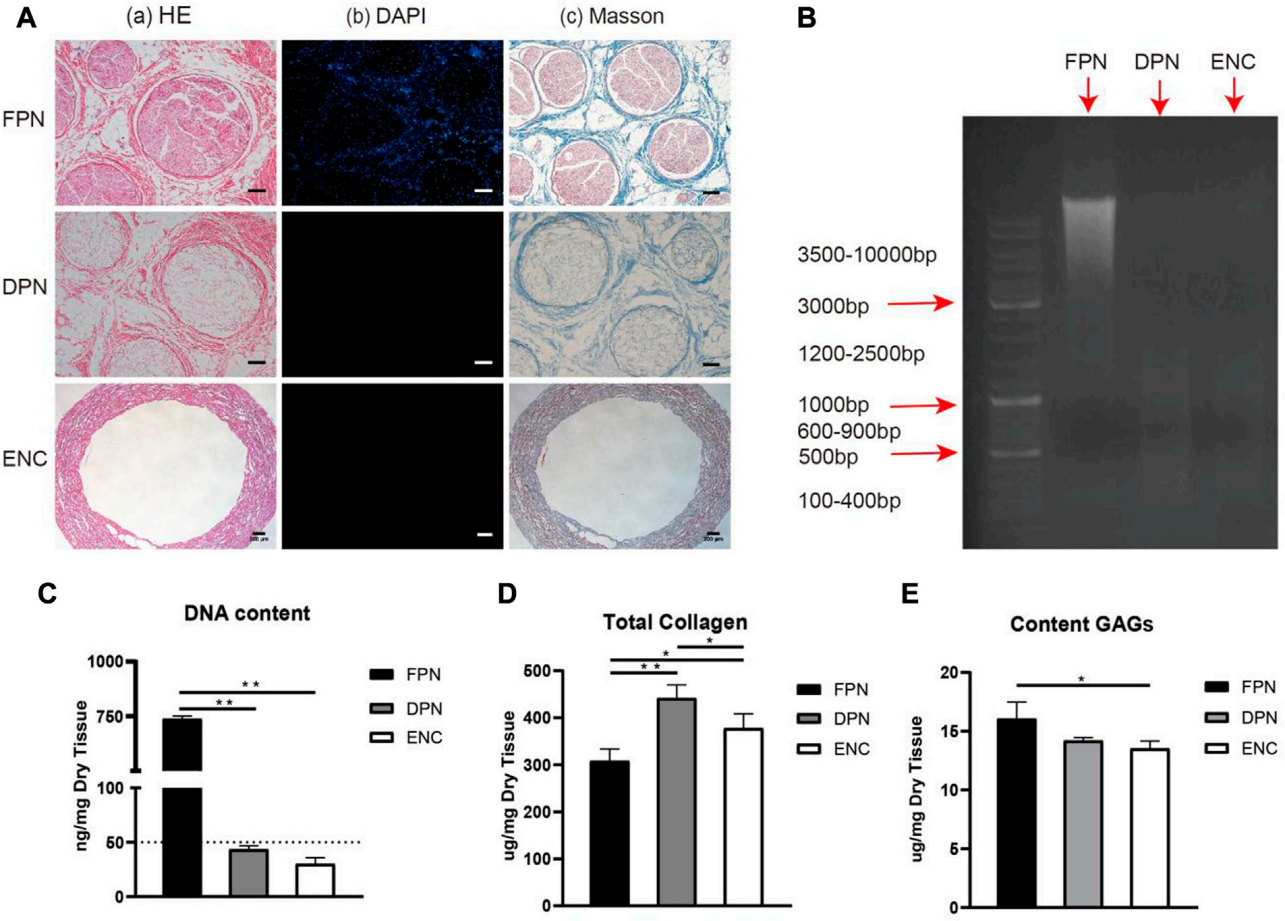
The evaluation of the extent of decellularization and the preservation of ECM components. The HE **(A–A)** and DAPI **(A–B)** staining were performed to evaluate the removal of cells. The Masson trichrome staining **(A–C)** showed the integrity and distribution of collagen after decellularization and electrospun. **(B)** The DNA remnants were analyzed further by agarose gel electrophoresis. **(C)** The detection of DNA content. **(D)** The detection of total collagen. **(E)** The detection of GAG. For FPN and DPN, scale bar = 100 μm, for ENC, scale bar = 200 μm. Data are expressed as the mean ± SD (*n* = 5) (*p < 0.05, **p < 0.01).

The images of porcine sciatic nerves before and after decellularization are shown in Figure 1A. After decellularization, the FPN changes from semitransparent to white and looks loosened. The HE and DAPI staining (Figures 2A-a, A-b) confirm that FPN shows a large number of dispersed cell nuclei. After decellularization, no visible cell nuclei in DPN can be detected as well as in ENC. The DNA quantitative assay (Figure 2C) suggests that the DNA amount is 738.30 ng/mg ± 12.58 ng/mg in dry tissue of FPN, while the amounts of residual DNA of DPN and ENC groups are 43.62 ng/mg ± 3.15 ng/mg and 30.09 ng/mg ± 5.69 ng/mg, respectively. The DNA quantitative assay suggests that both DPN and ENC show a significant reduction of DNA content compared with FPN (*p* < 0.05). The residual DNA contents of DPN and ENC have no significant difference (*p* > 0.05) and both of them are considered to meet the internationally recognized criterion of 50 ng/mg (Crapo et al., 2011; Wang et al., 2012b). Besides, the results of agarose gel electrophoresis reveal that there are no obvious bands of DNA (Figure 2B) in DPN and ENC compared to the whole genome bands in FPN. The results prove that our method realized sufficient decellularization.

### 3.3 Determination of total collagen and GAGs

To investigate the distribution and structure of collagen fibers within the samples of FPN, DPN, and ENC groups, sections are treated by Masson trichrome staining (Figure 2A-c). FPN exhibits a compact network of collagen fibers. In DPN, although cells and myelin sheaths are eliminated after decellularization, the arrangement of collagen fibers is still tight. In ENC, the arrangement of collagen fibers is regular porous.

As collagen and GAGs are the main components of ECM, their contents retained in FPN, DPN, and ENC are determined. Figure 2D shows that in dry tissue of the same weight, the contents of collagen are 308.50 μg/mg ± 24.96 μg/mg for FPN, 441.80 μg/mg ± 28.10 μg/mg for DPN and 378.00 μg/mg ± 30.58 μg/mg for ENC, respectively. DPN and ENC show an obvious increase compared with FPN (*p* < 0.05). The results indicate that the decellularization process and electrospun process do not remarkably destroy the collagen content. In the dry tissue of the same weight in FPN, DPN, and ENC, the GAGs contents are 16.07 μg/mg ± 1.42 μg/mg, 14.20 μg/mg ± 0.25 μg/mg, and 13.53 μg/mg ± 0.63 μg/mg, respectively (Figure 2E). The quantitative assay of GAGs indicates that ENC reveals a significant reduction compared with FPN (*p* < 0.05). Compared with FPN, GAGs content in DPN and ENC decreased, but there was no significant difference between DPN and ENC (*p* > 0.05). The results show that the electrospun process has no obvious effect on GAGs content.

### 3.4 Preservation of main ECM

Collagen I, collagen IV, LN, and FN construct the main components of ECM in FPN, which may be damaged during decellularization and electrospun processes. Immunohistochemistry, western blot, and ELISA are performed to measure these proteins.

**FIGURE 3:**
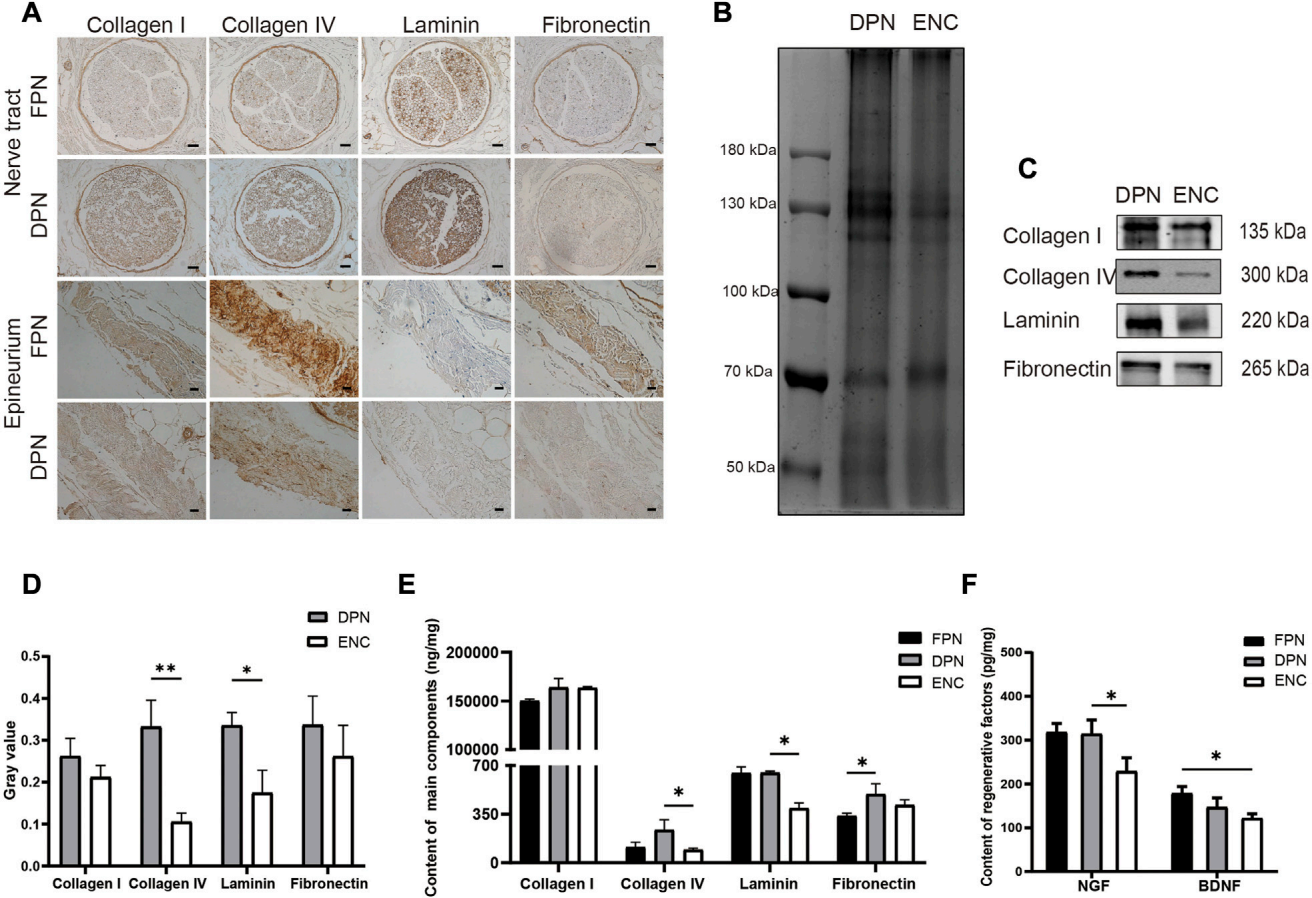
Comparison of main ECM bioactive molecules and regenerative factors in FPN, DPN, and ENC. **(A)** Immunohistochemical detection of collagen I, collagen IV, LN, and FN in FPN and DPN. **(B)** The total protein of the DPN and ENC groups was detected by Coomassie blue staining and used as a control for loading. **(C, D)** Collagen I, Collagen IV, LN, and FN were determined by western blot and quantified by grayscale analysis. **(E)** Collagen I, Collagen IV, LN, and FN were quantified by ELISA in FPN, DPN, and ENC. **(F)** NGF and BDNF were detected by ELISA. For the nerve tract, scale bar = 50 μm, for epineurium, scale bar = 20 μm. Data are expressed as the mean ± SD (*n* = 5) (*p < 0.05, **p < 0.01).

Immunohistochemical staining is used to detect collagen I, collagen IV, LN, and FN (Figure 3A) in FPN and DPN, which are located around the endoneurium and perineurium, and collagen I, collagen IV, and FN are found around the epineurium, and no LN is detected.

Western blot analysis is conducted to compare the preservation of these molecules in DPN and ENC (Figures 3B–D). Collagen I and FN in 2 groups are retained approximately at the same intensity (*p* > 0.05). However, for LN and collagen IV, the intensity of DPN group is higher compared with ENC (*p* < 0.05).

Furthermore, the quantitative detection of the contents of collagen I, collagen IV, LN, and FN are performed by ELISA. The contents of the 4 proteins in the total proteins of FPN, DPN, and ENC groups are shown in Figure 3E. The contents of FN increase significantly in the DPN compared with the FPN; however, there is no difference in the amounts of collagen I, collagen IV, and LN between DPN and FPN. Besides, the contents of collagen IV and LN are significantly decreased in the ENC compared to the DPN (*p* < 0.05). For collagen I and FN, there is no difference between DPN and ENC (*p* > 0.05).

NGF and BDNF are important factors that play vital roles in nerve regeneration. To measure the preservation of these factors in FPN, DPN, and ENC, quantitative detection is performed by ELISA (Figure 3F). The contents of NGF are significantly higher in DPN than those in ENC (*p* < 0.05), but there is no significant difference in the content of BDNF between DPN and ENC (*p* > 0.05). Additionally, there is no difference in the content of NGF and BDNF between FPN and DPN (*p* > 0.05).

### 3.5 Mechanical properties

**FIGURE 4:**
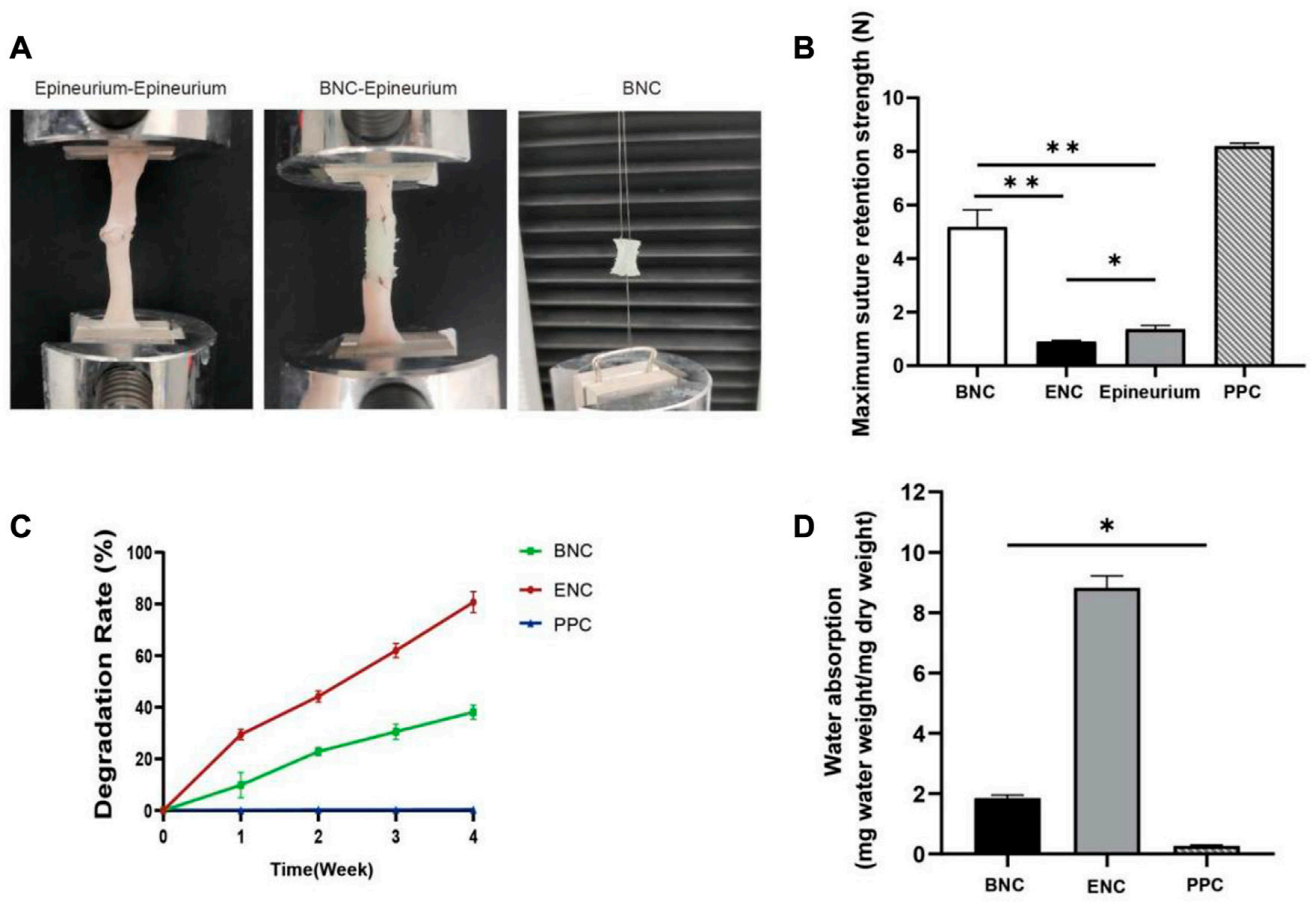
Assessment of the max suture retention strength. **(A)** Schematic representation of suture retention strength tested *in vitro*. **(B)** Suture retention strength was assessed to analyze mechanical properties of BNC, ENC, and PPC. **(C)** Weight change of BNC, ENC, and PPC during *in vitro* degradation in PBS at 37°C. **(D)** Water absorption analysis was performed to evaluate the effect of microstructure on the internal liquid exchange in BNC, ENC, and PPC. Data are expressed as the mean ± SD (*n* = 5) (*p < 0.05, **p < 0.01).

Adequate suture retention strength is required for BNC to maintain a mechanically robust interface with the native nerve stump during regeneration, which is critical for clinical use during surgical procedures. Therefore, in this study, the maximum values of the suture retention strength of the 3 conduits are tested and compared directly with fresh nerve epineuria (Figure 4A). The results are shown in Figure 4B, and for each of the conduits are 4.85 N (BNC), 0.89 N (ENC), and 8.19 N (PPC), respectively. From these results, we can see that PPC and BNC are significantly tougher than ENC (*p* < 0.05), and the suture retention strength of the BNC increases from 0.89 N to 4.85 N after electrospun PLA-PCL on ENC as the outer layer. In view of the reinforcement of PLA-PCL on the suture retention strength, in this study, it is necessary and appropriate to select and fabricate PLA-PCL to be the outer layer of BNC, so that to endow BNC with enough mechanical strength for surgical operation and implantation.

### 3.6 Degradability

The degradation rates of BNC, ENC, and PPC are shown in Figure 4C. The results show that with PLA-PCL as the outer layer of BNC, the degradation rate of BNC is significantly reduced compared with ENC (*p* < 0.05), which is 11.37% in the first week and reaches 28.30% in the fourth week. However, the degradation rate of ENC is nearly 80% in the fourth week, and ENC is almost completely degraded in the fifth week (data not shown). The degradation of PPC is negligible during this period.

### 3.7 Water absorption

To determine the effect of microstructure on the internal liquid exchange of 3 group conduits, the measurement of water absorption is performed (Figure 4D). The water absorption of ENC and BNC increases significantly compared to PPC (*p* < 0.05), and ENC is significantly higher than BNC (*p* < 0.05).

### 3.8 Cytotoxicity assay

**FIGURE 5:**
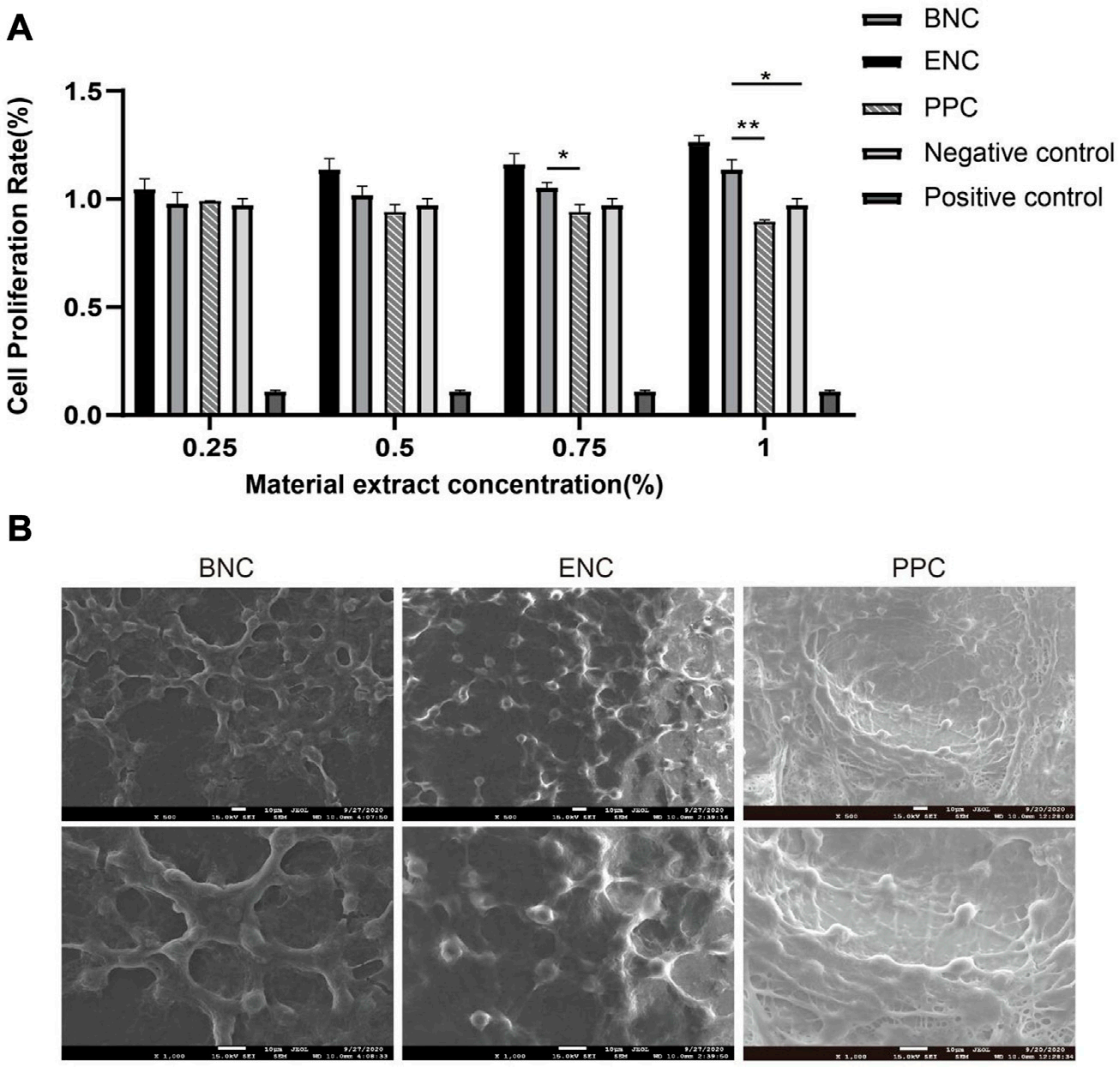
Comparison of CCK-8 assay and biocompatibility of BNC, ENC, and PPC. **(A)** The trend of cell relative proliferation rates of BNC, ENC, and PPC at different concentrations was observed on the third day. **(B)** SEM images of SCs adhesion on the surface of BNC, ENC, and PPC. Data are expressed as the mean ± SD (*n* = 5) (*p < 0.05, **p < 0.01).

The CCK-8 assay is performed to determine the cytotoxicity of the 3 group conduits. After 1, 3, and 5 days of cell culture, the extracts of BNC, ENC, and PPC at different concentrations are observed for OD_450_ values. The results of previous experiments show that when the density is 2 × 10^4^ cells/well, the cell proliferation rate reaches the maximum on the third day, so the third day is selected as the time point to discuss the effect of different concentrations of extracts on the cell proliferation rate. And the results are presented in Figure 5A. The results suggest that the BNC and ENC extracts not only have no toxic effect on SCs, but also play a positive role in cell proliferation. Furthermore, the cell proliferation rates of BNC and ENC extracts are significantly higher than that of the negative control groups with increasing concentrations (*p* < 0.05) and reach the maximum value at the extract concentration of 100%. The higher the extract concentrations of BNC and ENC are, the better the cell viability is. In addition, although the PPC extracts do not prompt cell proliferation, it has no toxic effect on cells.

### 3.9 SEM observation of cell affinity

SEM shows that SCs have already adhered to the surface of the 3 groups of membranes derived from conduits. As shown in Figure 5B, SCs ultimately spread all over the membranes, and form a cell layer on the surface of the membranes. The morphology of SCs on the PPC membrane is atypical and mostly rounded, whereas the SCs on the BNC and ENC membranes present bipolar spindle bodies, with two more than elongated protrusions, a few show triangles or polygons, and fused into slices. The number of SCs adhering to the membrane surface of BNC and ENC is more than that of PPC. Meanwhile, SCs on BNC and ENC membranes have a more regular arrangement than on PPC membrane. These observations indicate that BNC and ENC have better biocompatibility than PPC, which supports cell attachment and proliferation.

### 3.10 Subcutaneous implantation tests

**FIGURE 6:**
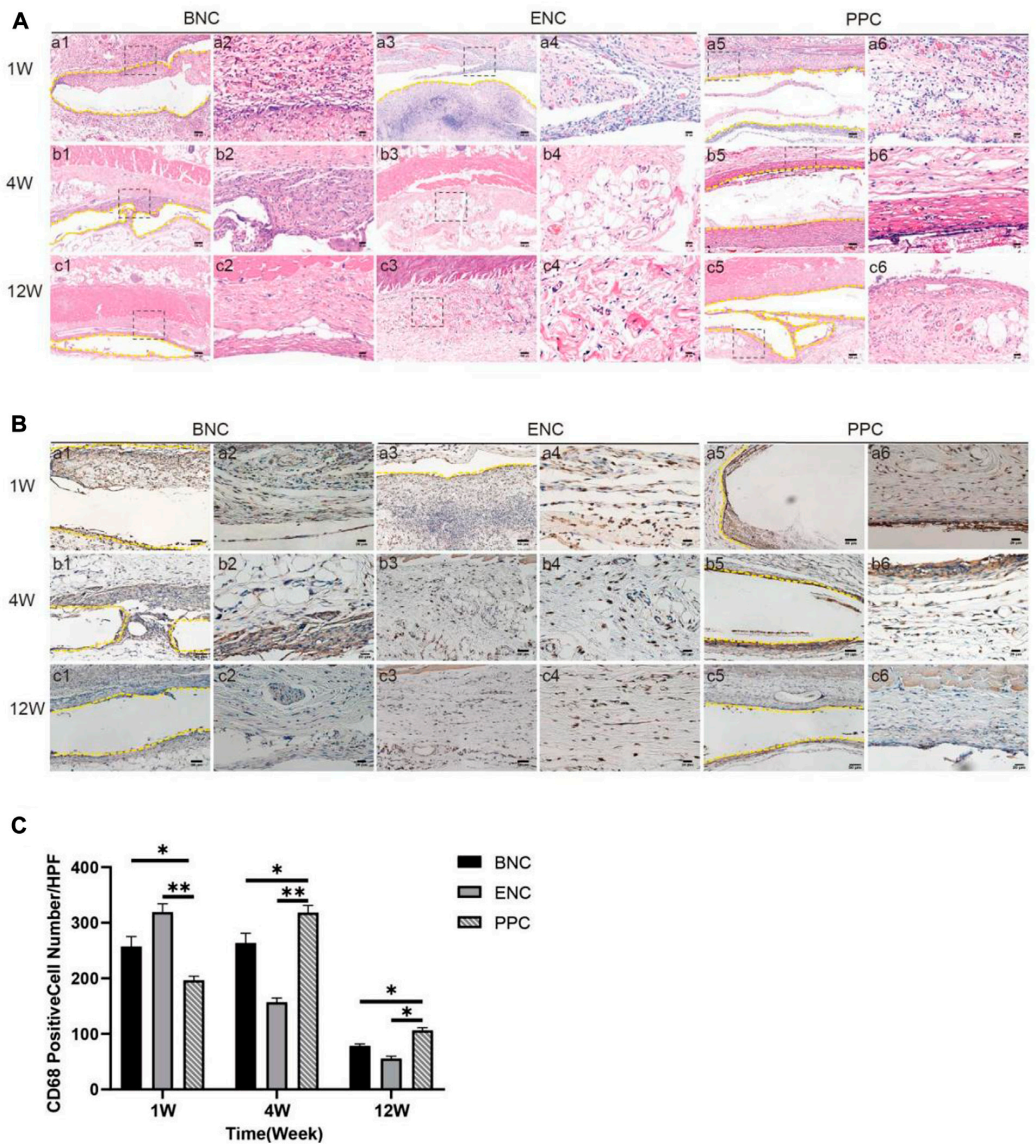
Evaluation of the host immune response to the BNC, ENC, and PPC groups. **(A)** Histological observation of the subcutaneous implantation tests by HE staining. It shows the type and number changing processes of inflammatory cells in 1, 4, and 12 weeks after implantation. Images with higher magnification (× 400) (scale bar = 20 μm) represent the area within the black box in lower magnification images (× 100) (Scale bar = 100 μm). **(B)** Histological observation of the subcutaneous implantation tests by immunohistochemical staining. It shows the number and distribution of CD68 macrophages in the 3 groups during the healing process. Images with higher magnification (× 400) (scale bar = 20 μm) represent the positive area of the surrounding tissue at the edge of the material. The yellow dotted line in the images with magnification (× 200) (scale bar = 50 μm) is the place where the graft is placed. **(C)** Quantification of immunohistochemical staining by positive cell counting at × 400. Data are expressed as the mean ± SD (*n* = 5) (*p < 0.05, **p < 0.01).

HE staining is conducted to demonstrate the changes in the number and distribution of inflammatory cells after subcutaneous implantation (Figure 6A). In the first week, a big quantity of inflammatory cells infiltrate around the implanted 3 group conduits, while there is no obvious difference between the 3 groups. In the fourth week, increasing inflammatory cells are observed aggregating towards the implanted BNC and PPC region, and most of them are mononuclear macrophages. In addition, the BNC membrane degraded obviously, and the ENC membrane degraded completely. Till week 12, there are almost no inflammatory cells left in or around BNC and ENC membranes, however, there are still inflammatory cells flocking together around PPC membrane.

The immunohistochemical images mainly show the role of CD68-positive macrophages in the implantation process of 3 group membranes (Figure 6B). In the first week, a large number of CD68-positive cells begin to appear in the adjacent tissues to the membrane implanted in each group, and the quantitative analysis of the CD68-positive cell immunohistochemical image (Figure 6C) shows the numbers of CD68 in BNC and ENC groups are significantly higher than those in PPC group (*p* < 0.05). In the fourth week, CD68 mononuclear macrophages are the dominant cells around the materials in each group, meanwhile, the number of CD68 in PPC is significantly higher than in BNC and ENC groups (*p* < 0.05). Until the twelfth week, in all groups, the number of CD68-positive cells decreases, which is consistent with the trend of at the fourth week (*p* < 0.05).

### 3.11 Functional recovery evaluation

**FIGURE 7:**
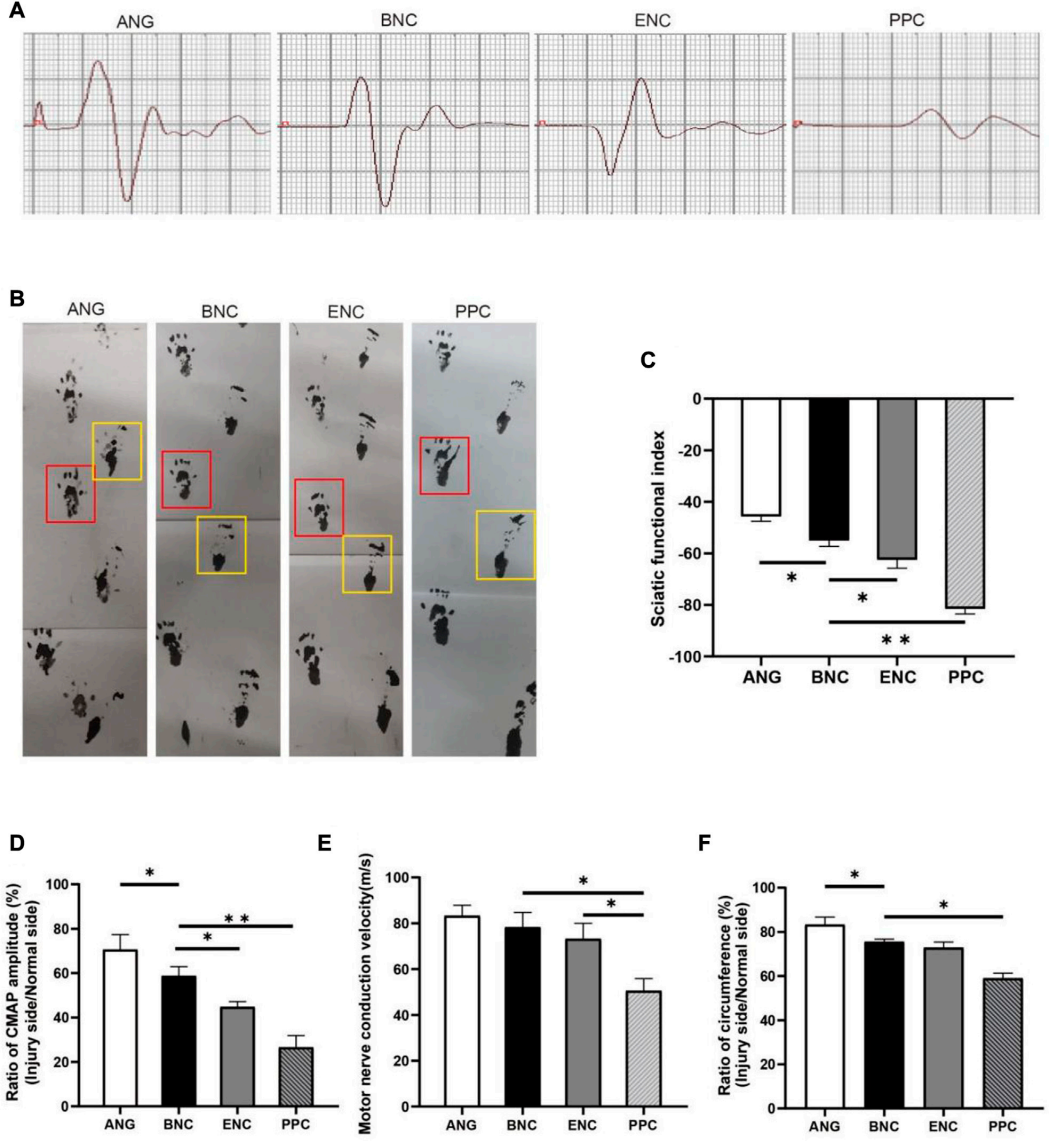
The evaluation of electrophysiology and SFI values of regenerated nerves. **(A)** Representative CMAP recordings at the injury site. **(B)** Footprint analysis in each group (the yellow box represents the injured side; the red box represents the normal side). The histograms show SFI value **(C)**, ratio of CAMP amplitude **(D)**, MCV **(E)** and, ratio of circumference **(F)** at 12 weeks after surgery. Data are expressed as the mean ± SD (*n* = 5) (*p < 0.05, **p < 0.01).

SFI is an indicator of motor function recovery following sciatic nerve injury, an SFI value of 0 indicates normal motor function, whereas a value near −100 represents complete dysfunction (Bain et al., 1989) (Figure 7B). The initial SFI values of each group are plunged to near −100 and then are improved with time. Each group’s final SFI is recorded in Figure 7C as follows: ANG group (−45.67 ± 1.95), BNC group (−54.99 ± 2.32), ENC group (−62.50 ± 3.29), and PPC group (−81.48 ± 2.07), respectively. The SFI of the BNC group is significantly higher than those in ENC and PPC groups (*p* < 0.05), but lower than those in the ANG group at 12 weeks.

### 3.12 Muscle recovery evaluation

The circumference ratio between the injured side and normal side can represent the degree of muscle recovery. As depicted in Figure 7F, the circumference ratios in BNC and ENC groups are significantly larger than those in PPC group (*p* < 0.05), but there is no significant difference between BNC and ENC groups (*p* > 0.05).

### 3.13 Electrophysiological evaluation

Electrophysiologic assessment is performed to evaluate the functional recovery after 12 weeks post-surgery, records from gastrocnemius muscle followed by measurements of the ratio of CMAP amplitudes and MCVs. Each group’s CMAP amplitude is recorded in Figure 7A, the ratio of CMAP amplitude (Figure 7D) between the injured side and normal side in the BNC group (70.62% ± 6.75%) is significantly higher than those in the ENC group (44.75% ± 2.42%, *p* < 0.05) and PPC group (26.57% ± 5.28%, *p* < 0.05), and lower than the ANG group (70.62% ± 6.75%, *p* < 0.05). In addition, the results of MCVs show that MCVs in the BNC (73.21 m/s ± 6.76 m/s) and ENC groups (78.33 m/s ± 6.38 m/s) are significantly higher than those in the PPC group (50.57 m/s ± 5.29 m/s, *p* < 0.05), but there is no significant difference between BNC and ENC groups (*p* > 0.05) (Figure 7E). According to the electrophysiological results, the MCVs and CMAP amplitudes of the BNC group are significantly higher than those of the PPC group (*p* < 0.05), but lower than those of the ANG group at 12 weeks after surgery. These results indicate that the ECM components promote re-myelination and facilitate the electrophysiological recovery of the regenerated nerve.

### 3.14 Toluidine blue staining assessment

**FIGURE 8:**
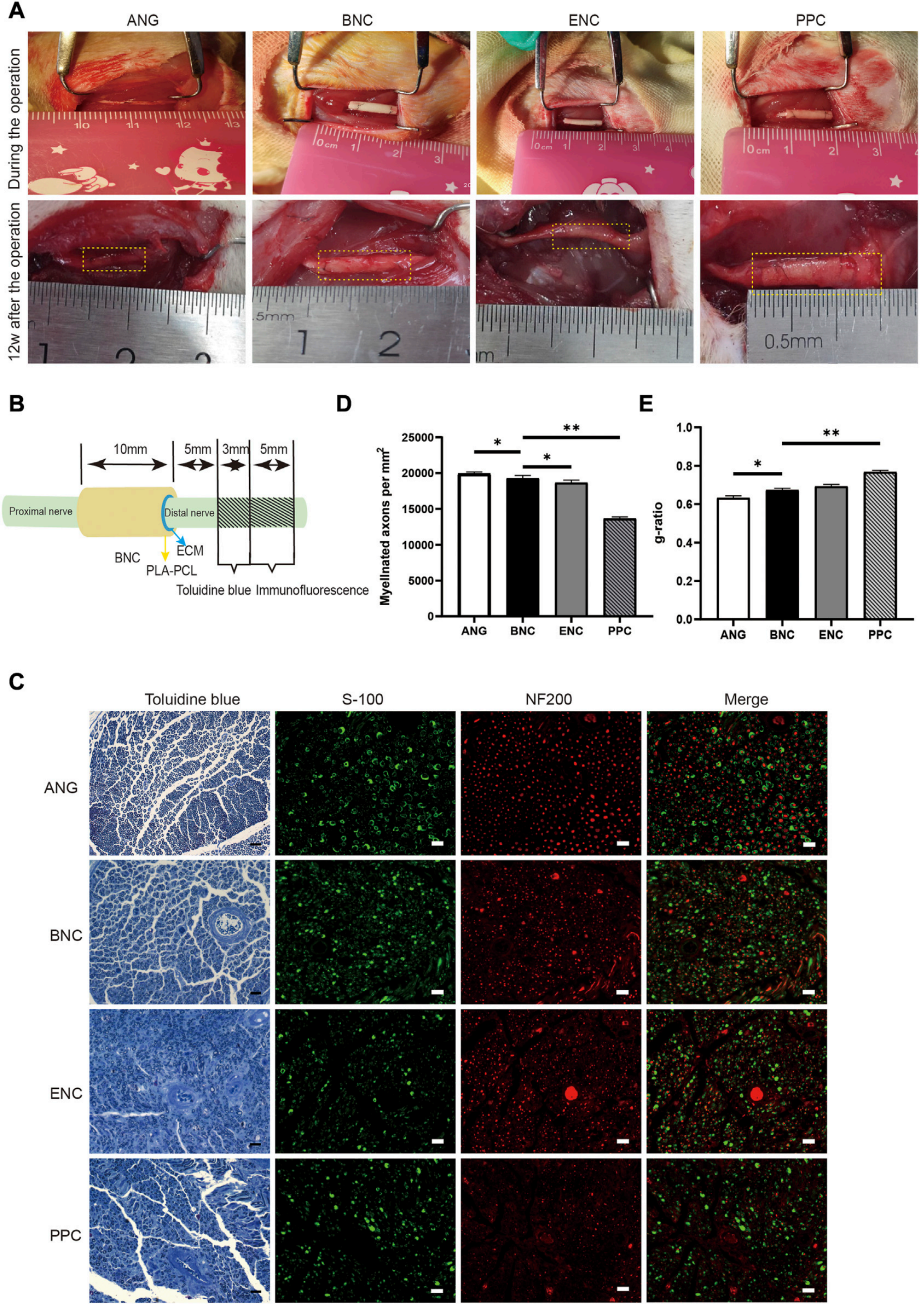
Implantation, observation after the operation, and the assessment of the regenerated axons and myelin sheaths in different groups. **(A)** The conduits implanted in 10-mm rat sciatic nerve defect and observed at 12 weeks after surgery. **(B)** Schematic diagram of the sampling locations for toluidine blue staining and immunofluorescence assay. **(C)** Toluidine blue staining of the regenerated myelin sheath at 12 weeks after surgery. Scale bar = 20 μm. **(D)** Histogram of the myelinated axon numbers/HPF at 12 weeks after surgery. **(E)** Histogram of the g-ratio at 12 weeks after surgery. Data are expressed as the mean ± SD (*n* = 5) (ns > 0.05, *p < 0.05).

12 weeks after surgery, to evaluate the ability of each group of conduits to promote nerve regeneration, the cross section of the distal segments near the conduits site and ANG is observed by toluidine blue staining of the myelin sheath. The numbers of myelinated axons in each group are shown in Figure 8D, the number of the myelin sheath is 19,941 ± 224.7 for ANG group, 19,280 ± 403.0 for BNC group, 18,662 ± 374.3 for ENC group, and 13,642 ± 241.9 for PPC group, respectively, which reveal that the numbers of myelinated axons in the BNC group and ENC group are significantly higher than those of the PPC group (*p* < 0.05), but there is no significant difference between BNC and ENC (*p* > 0.05). As Figure 8E shown, the g-ratio is 0.633 ± 0.011 for ANG group, 0.674 ± 0.009 for BNC group, 0.693 ± 0.010 for ENC group, and 0.768 ± 0.008 for PPC group, respectively, which prove that the maturation of fibers and their speed of conduction in the BNC group are significantly higher than those of the ANG and PPC group (*p* < 0.05), but there is no significant difference between BNC and ENC (*p* > 0.05).

### 3.15 Immunohistochemical studies

12 weeks after surgery, the cross-sections of the nerve distal segments near the end of the nerve conduits from each group are stained by immunofluorescence assay for the regenerated axon (NF200) and myelin sheath (S100). As shown in Figure 8C, the histological recoveries of the regenerated nerve tissues from the BNC group are better than those from the PPC group and are close to the gold standard ANG group.

## 4 Discussion

During the last decade, ECM materials have been widely acknowledged as advanced functional biomaterials in regenerative medicine (Szynkaruk et al., 2013; Wang et al., 2020; Yu et al., 2022). Successful removal of cells and DNA from tissues is a pre-requisite for the biomaterials derived from xenogeneic mammalian models. Excessive residual cells and DNA have the potential to cause inflammatory responses, which can lead to the rejection of the graft *in vivo* (Keane et al., 2012). Our previous study reported a method by which porcine peripheral nerve has been successfully decellularized with low DNA content and primary ECM components including collagen, LN, and FN retained (Zilic et al., 2016). Another study proposed that the DNA content of a decellularized graft should be less than 50 ng/mg, and there should be no visible nuclei in tissue sections (Crapo et al., 2011).

In the current study, the electrospun nanofibrous web of PLA-PCL and ECM was used to fabricate a bilayer structured nerve conduit (BNC), which combined the advantages of PLA-PCL nanofibers including flexibility and suitable mechanical properties, and good cellular affinity of ECM, which are the essential requirements for neural conduits. Moreover, it can be found that no remnant cell nucleus can be detected in DPN and ENC, and the residual DNA contents of DPN and ENC have dramatically decreased to less than 50 ng/mg without DNA fragments larger than 200 bp. These results demonstrate that the adopted decellularization method can eliminate cellular components effectively, which ensures low immunogenicity.

Collagen I, collagen IV, LN, and FN are very important for the development and regeneration of the nervous system, and play key roles in regulating the proliferation, differentiation, and function of SCs (Gu et al., 2014; Chang et al., 2017; Quraishe et al., 2018). The mammalian neurotrophins (NTs), a family of structurally-related proteins, regulate neurite outgrowth and modulate neuronal differentiation and survival (Thomaz et al., 2020). Among the family of NTs, BDNF and NGF are small proteins expressed in the brain and peripheral tissues, which have been studied extensively and play important roles in neuronal survival, differentiation, neurogenesis, and synaptic plasticity (Nordvall et al., 2022). Therefore, we have chosen these bioactive molecules as our research targets. To compare the preservation of main bioactive molecules in ECM, immunohistochemistry was used to detect collagen I, collagen IV, LN, and FN in FPN and DPN but not ENC. ENC loses the physical structure of DPN with various ECM components in a mixed arrangement, so it is impossible to detect the main position of these bioactive molecules in ENC. In the present study, western blot is utilized to analyze the difference between DPN and ENC in ECM bioactive molecules. It is worth noting that the FPN group is not introduced as a control group, and the reason is that in western blot, when the total protein content of each group is equal, the presence of cell protein leads to the increase of relative content, and the expression level of bioactive components in FPN is very low, lower than that in DPN. To avoid misleading conclusion, this study directly compared the relative content of bioactive components in DPN and ENC groups and analyzed the effect of the electrospun process on the preservation of ECM. The results show that ENC not only retained collagens, LN, and FN effectively, but also preserved BDNF and NGF. It is noted that the total collagen contents of DPN and ENC are even higher than that of FPN (Figure 2D). This is because the total collagen content is expressed as the proportion of the dry weight of tissue. After the removal of cell components, the significant decrease in tissue dry weight resulted in a higher proportion of total collagen content in DPN and ENC (Philips et al., 2018).

On the other hand, the remaining GAGs in the ENC could participate in the regulation of axonal growth (Lee et al., 2014). The content of GAGs in DPN and ENC both decrease significantly, but the GAGs content of ENC is 84.19% of the content in FPN. Additionally, GAGs could partly promote the polymerization of collagens in ECM solution, which could contribute to the formation of the ENC and support its mechanical properties (Stuart and Panitch, 2008).

Neural scaffolds demand adequate mechanical properties to support regenerative axons and myelin sheaths, therefore it is essential to analyze from a biomechanical perspective. Suture retention strength related to epineurium is crucial to maintain the coaptation between conduit interfaces and nerve stumps (Ao et al., 2006; Yu et al., 2020). In this study, there is a significant increase in the max suture retention strength of BNC compared with native epineurium. The max suture retention strength of the BNC is also higher than that of ENC, which indicates that the suitable diameter of PLA-PCL nanofibers increased the suture retention strength.

The ability to support the survival and biological behaviors of SCs is an important consideration and a basic requirement for nerve repair materials (Kehoe et al., 2012). In this study, the results of the biocompatibility evaluation reveal that BNC and ENC could support the growth and proliferation of SCs and exhibited good biocompatibility (Figure 5B). Polymer blending (usually with natural polymer) is one of the several efforts used to enhance the hydrophilicity of the electrospun nanofibers (Neal et al., 2014). Some ECM proteins, including collagens and FN, are necessary for the growth and motility of SCs (Yang et al., 2011; Szynkaruk et al., 2013). In addition, the nanofibrous structure of ECM consists of a network of nanofibers, this structure probably promotes cell adhesion and differentiation, as well as the absorption of nutrients and active factors (Xie et al., 2010; Zeng et al., 2014; Nune et al., 2016). Therefore, the presence of these ECM proteins and the nanofibrous structure of ECM make BNC an ideal biocompatible nerve conduit. Meanwhile, the CCK-8 test is performed and the results indicate that the extracts of BNC not only have no toxic effect on the cells, but benefit cell proliferation to some degree.

A 10-mm rat sciatic nerve defect model has often been used to evaluate the effect of artificial nerve conduits for nerve regeneration (Sun et al., 2017). Therefore, in this study, we created a 10-mm gap in the rat sciatic nerve, and in functional evaluation assays, the sciatic functional index is calculated to assess the functional recovery of the motor nerve after nerve injury. After 12 weeks, the electrophysiological test shows that the MCV in the BNC and ENC groups are similar. MCV is positively correlated with the number of myelinated nerves crossing the gap and/or axon diameter (Chang et al., 2017), which is further confirmed by the toluidine blue staining. The result of MCV implies that the BNC supports more nerve fibers to grow across the gap quickly and re-innervate more target muscles than the PPC group, which suggests that due to the co-existence of PLA-PCL and ECM in BNC, BNC may improve the recovery of nerve function by extending the degradation time and increasing ECM related proteins. It is worth noting that the results of MCV and myelin sheath numbers show no significant difference between the BNC group and ENC group, which may be because the length of the nerve defect is too short in the study so that the nerve defect has been fully combined within 4 weeks (the degradation time of ENC). The affected area of rats is not seriously impacted by the outside world during feeding. Also, there was no rupture in ENC before neurorrhaphy. The immunofluorescence histopathology analysis confirmed the significant migration and proliferation of SCs from the proximal to the distal stump of nerve fibers, which indicated that the presence of ECM improved the biological activity of the BNC.

Although the positive results of the *in vitro* and *in vivo* studies demonstrated that the BNC had the potential for clinical application, some important bioactive molecule contents were reduced or even lost during the manufacturing process. This shortcoming could be compensated by adding biological molecules like growth factors and small regenerative molecules into the inner layer (ECM) of BNC to achieve the desired outcomes of nerve regeneration and functional recovery in the possibly shortest time (Allen et al., 2013; Gao et al., 2016). Meanwhile, electrospun nerve conduits can be easily used for loading with growth factors and other regenerative molecules (Dalamagkas et al., 2016). In addition, this potential will be enhanced by optimizing decellularization technology, retaining as many active components as possible, or improving the manufacturing technology of the BNC, thereby facilitating the reconstruction of the biological microenvironment to enhance nerve regeneration (Houschyar et al., 2016; Yu et al., 2020). Overall, the BNC outer-inner layer structure prepared by the electrospun method exhibited high potential in regenerating defected sciatic nerve both *in vitro* and *in vivo,* and would be a good candidate for further investigations.

## 5 Conclusion

In this study, a bilayer nerve repair conduit composed of ECM and PLA-PCL were prepared by electrospun technique for the first time, and proves suitable for peripheral nerve repair. The outer PLA-PCL layer of BNC is effective in prolonging the degradation time, and enhancing the mechanical properties of the conduit, and the inner ECM layer improves the biocompatibility and bioactivity of the conduits. Therefore, the BNC has great potential for clinical repair of nerve defects.

## Data Availability

The raw data supporting the conclusion of this article will be made available by the authors, without undue reservation.
